# A profile of South African sustainable animal production and greenhouse gas emissions

**DOI:** 10.1093/af/vfab025

**Published:** 2021-09-06

**Authors:** Norman H Casey

**Affiliations:** Department of Animal Science, University of Pretoria, Private Bag X20, Hatfield 0028, South Africa

**Keywords:** agriculture policies, livestock emissions, livestock systems, sustainable agriculture, livestock legacy

**Implications**

- South Africa has a precolonial livestock production legacy that is carried through to modern times.
- The livestock industry through its structural organizations, endorsed the country’s commitments to the UNFCCC (1997), the Kyoto Protocol (2002), and the Paris Agreement on Climate Change (2016) to actively manage greenhouse gas emission (GHGe), along the producer-to-consumer value chain, and to promote sustainable animal production practices.
- The genetic base of the livestock industry is enriched by a combination of original indigenous and exotic cattle, sheep, goats, chickens, and swine in combination with exotic genotypes.
- The livestock industry and government actively support developing sustainable animal production systems aimed at reducing GHGe.
- The South African livestock industry acknowledges and protects the original, indigenous livestock whose adaptive traits have been incorporated in composite breeds as a component of promoting sustainable livestock production and managing GHGe emissions.

## Introduction

South Africa’s rich legacy of sustainable animal production reaches back centuries. Extensive animal production with cattle, sheep, and goats in pastoral systems and chickens and swine around settlements was well established among the African inhabitants of southern Africa before the European exploration and settlements. Animal production had migrated down Africa from the Middle-East and North Africa.

Cattle were present in the Zambezi region 300 BCE and small stock at the southern tip of Africa, the Cape of Good Hope, 20 centuries ago (Maree and Plug, 1993; Mason and Maule, 1960). The presence of a sustained animal industry in southern Africa for millennia emphasized that pastoral systems fit in well with the wildlife that populated the region. The inhabitants drew sustenance from both domesticated and nondomesticated animal clusters and were sustainable despite sporadic droughts and endemic livestock diseases. The livestock were physiologically and morphologically highly adapted to the range of climatic regions and the seasonal variation of the nutritional value of natural herbage. Southern Africa with its indigenous livestock and inherent animal production practices was uniquely different from the Americas that had few domesticated animals (Stahl, 2008) and Australasia that had none (Parsonson, 2000) at the time of the European colonization. The region had the intellectual capital for livestock farming with suitable types of livestock.

This article presents a profile of sustainable animal production and greenhouse gas emissions against an historical background, and the gains in animal husbandry in South Africa, which are important in the long-term context of sustainability.

## Historical Background

The livestock heritage enabled a relatively quick transition from pastoralism to settled farming. Prior to European colonization, the land was held through conquest and agreements by indigenous people clustered in tribes and family units within tribes. Grazing rights, or land-usage between tribes, was often settled belligerently, and within tribes and among family units, by tribal customs overseen by headmen or women according to patriarchal or matriarchal societies.

The Dutch established a replenishment post at the Cape of Good Hope that became Cape Town in 1652 that began a steady flow of Europeans to southern Africa. The immigrants brought their legal culture, which became the norm, excluding the regions beyond the colony’s borders. Among these was land ownership.

Growing requirements for services by passing tradeships and the increasing population in Cape Town of Europeans, imported slaves and servants, and indigenous African people, stimulated the need for food and services. In the 1690s, French Huguenot refugees increased the European population. The authorities came under pressure to extend the boundaries of the settlement and lay out farms.

The land appeared to be largely uninhabited. Nomadic hunter-gatherer people commonly referred to as Bushman now recognized as the San people (SAHO), lived off the land. The Khoikhoi people were nomadic herders within distinct tribal boundaries along the southwestern regions with cattle, sheep, and goats (SAHO). They were the first inhabitants with livestock that the Dutch had encountered and traded. The pressure for land and expansion had caused the Cape government to obtain land from the Khoikhoi through bartering and agreements. Initially, farms were held under lease agreements from the Cape government that were later converted to freehold properties with title deeds. These farms became the centers for animal production relying on the local indigenous animals.

Many settlers became restless for political and economic reasons and set out trekking beyond the settlement’s borders into the hinterland as pastoral farmers. Economics was an important stimulus because the settled farms had clashed head-on with nonsustainability. By becoming itinerant graziers, that immediate problem could be resolved. Government found little comfort in a bunch of itinerant farmers moving around and clashing with the African people. The boundaries of the colony were extended, farms laid out, and taxes levied.

The movement of people from the colony spread further with more land being attained from African tribes through treaties and trade-off, coercion, and belligerence. In the end, freehold of the land was abundantly in the hands of White people. The indigenous Khoikhoi people in the western regions and the Nguni Africans in the northern and eastern regions remained tribal bound. The African tribal areas were recognized and referred to according to the principal tribe of the regions, hence Zululand, or to the traditional paramount chief of the tribe, such as Sekhukhune-land (SAHO).

## Segregation’s Impact on Optimizing Sustainable Animal Production

Racial segregation, nonofficial and official, and civil rights and thereby land-use were instituted in various forms during the colonizing period, which extended to 1994. The Cape Colony Government, under the oversight of the British Colonial Office, passed the Glen Grey Act (1894) that established systems of land tenure and labor (Thompson, 1991). The strict Apartheid system of separation, or separate development as it was euphemistically referred to, began with the Population Registration Act (1950) by which people were officially racially classified. The Apartheid system was systematically built through a series of legislation including the Black Administration Act (1927) (www.gov.za), the Bantu Trust and Land Act (1936) (www.gov.za), the Promotion of Bantu Self Government Act (1959) (www.gov.za), and the National States Citizen Act (1970) (www.gov.za). Africans were to become citizens of a self-governing territory, thereby forfeiting citizen rights in the rest of the Republic. Within these territories, land-use rights were allocated principally according to custom with few freehold properties. In a process of consolidating traditional tribal regions into self-governing territories, the government incorporated registered farms bought from commercial farmers.

Due to this long process of separation, the animal industry across South Africa had emerged into two distinct forms: Freehold among the Whites and some Khokhoi families and immigrant Indians of the KwaZulu-Natal region; the majority of Nguni Africans were in traditional communal land-use structures with a sprinkling of freehold farms.

Although much of the Apartheid legislation has been repealed since its abolition, the consequences prevail. Restrictions on land ownership were canceled, which opened opportunities to the broader community. Only a few have had the means to buy farms, while traditional land allocation and use have continued. The Restitution of Land Rights Act (1994) was introduced (www.gov.za). Attaining the envisaged successes has lagged due to a complexity of reasons, however. These include institutional obstacles (Walker, 2012), the lack of farming and business capabilities, and socioeconomic factors such as limited access to developmental capital, and traditional customs. Restitution of agricultural land and associated business economic imperatives (Binswanger-Mkize, 2014) are critical toward attaining optimized sustainable animal production.

## Sustainable Animal Agriculture

Sustainability is a broad concept with five key constructs, adapted from Moore et al. (2017): A defined period of time, continued delivery intervention strategies, maintaining behavioral change, and adapting the program and behavior while continuing producing benefits for the system. Sustainability is essentially a nonself-perpetuating system requiring drivers in the industry. It is an open, dynamic state for which variables can change and either advance or impair it. An inclusive definition for sustainable agriculture could be “agriculture that can evolve indefinitely toward greater human utility, efficiency of resource use, and a balance with the environment that is favourable both to humans and to most other species” (Harwood, 1990), and within norms of ethical practices (Webb, 2013). Sustainability is not an end, but a progression. It embodies efficient resource-use and human capital, advancing genetics, nutrition and health management, structured and balanced economic systems, optimal natural resources management, and an enabling political environment. Efficiency is overarching; striving for sustainability is striving for efficient production.

### Overview of Sustainable Animal Agriculture

Utilizing the extensive rangeland with cattle, sheep, and goats is the main driver of the livestock industry producing a range of products, and sustains vast numbers of employees and dependents (Meissner et al., 2013a, 2013b).

Commercial agriculture occupies 38% of the total land area. Animal agriculture occupies 79% of the agricultural land and employs >21% of the agriculture workforce (Statistics SA, 2020). Agriculture maintained a steady 2.3% of GDP, with industry contributing 26.6%, and services 71.2% (Plechter, 2020). The sector maintained 47% against field crops, 44.3%, and horticulture, 22.8% (Vink and van Rooyen, 2009).

In the extensive production sector, the prospect of growth in beef production would rely on improving production rates, while the small stock industries (wool, meat, mohair, and karakul) have limited growth potential under current production practices. Economic conditions constrain growth in the dairy sector. The swine industry has limited growth prospects due to a sectoral consumer profile. The chicken industry (eggs and meat) has the greater growth prospects, though it currently faces stiff competition from imported products. National sustainable animal production would gain by successful land restitution and transformation of traditional African tribal lands to units driven by business economic imperatives (Binswanger-Mkize, 2014).

South Africa has 12.8-m cattle, 19.4-m sheep, 3-m goats, 1.5-m swine (DALRRD, 2020), and 38.2-m poultry (layers 27.6 and broilers 10.6) (SAPA, 2019). Animal production’s gross value is 46% of the agricultural gross value (Table 1). Within the sector, bovine products are 40%, poultry meat and eggs 39%, followed by ovine and caprine products, 10%.

**Table 1.** Gross value of South African agricultural products in 2016

Sector	Gross value (ZAR’000)	Gross value (%)
Field crops	60,598,718	23.3
Horticultural products	79,043,004	30.4
Animal products	120,128,788	46.2
Animal products by sector	Gross value (ZAR’000)	Animal products (%)
Wool, Mohair, and Karakul pelts	4,038,902	3.4
Ostrich products	438,903	0.4
Poultry meat	36,669,836	30.5
Eggs	10,191,731	8.5
Cattle and calves slaughtered	33,003,889	27.5
Sheep and goats slaughtered	7,158,715	6.0
Swine slaughtered	5,566,721	4.6
Milk	15,659,645	13.0
Other animal products	7,400,446	6.2

Source: DALRRD (2020).

The animal industries’ sectors are structured as extensive, semi-intensive, and intensive production systems. Extensive primary production systems are beef, wool and meat, and mohair producing goats. A small, variable Karakul industry functions within the sheep cluster producing pelts and meat. Large numbers of beef cattle and a few sheep systems flow over to intensive feedlots for finishing animals to market weight following the weaning period. The dairy industry uses a combination of intensive to semi-intensive production. Swine and poultry sectors are highly industrialized systems.

Extensive systems use the natural range, valuable resources that is mostly nonarable land. In addition, the animal manure and urine are distributed over the range entering the natural carbon cycle, whereas the methane and carbon dioxide constitute recycling carbon. Extensive production, however, requires additional services that could contribute to the carbon footprint, which include veterinary support, supplementary feed where necessary such as licks, transport, electricity if not off-grid, and general goods and services.

Semi-extensive dairy systems use natural range or established pastures for cows in milk, dry cows, heifers, and often bulls. Intensive dairy production, feedlot feeding beef weaners, and swine and poultry production systems are entirely dependent on off-farm produced feed, supporting services and mechanized infrastructure. If one accepts that all animals use plant-based feeds and therefore recycling carbon, the net carbon footprint is ascribed to direct and indirect supporting services.

## Developing Sustainability

Sustainability requires continual reassessment of animal productivity, product quality, rangeland and supplementary feeding resources, water, and marketing channels. Hardy indigenous cattle, sheep, and goats did not fulfill the productivity expectations or the required quality of the products. Two actions were launched: Improving genetics and recording productivity. Breeds were imported to either replace the indigenous animals or to crossbreed to improve productivity and product quality. Imported livestock became indigenous by virtue of their adapted physiology and morphology, selection for productivity traits, and by crossbreeding with indigenous livestock. The officially recognized indigenous livestock (Table 2) are either the original types or developed types based on the original types often with exotic genotypes incorporated. Examples are the composite Bonsmara cattle breed and the Dorper meat sheep breed. The imported Persian and Karakul sheep are considered to have the correct attributes for farming in South Africa’s harsh, semidesert regions.

**Table 2.** Indigenous breeds of cattle, sheep, goats, swine, and poultry

Cattle	Sheep	Sheep	Goats	Swine	Poultry
Original	Original	Developed	Original	Original	Original
Afrikaner	Afrikaner	Afrino	Savannah	Black indigenous	Naked Nek
Nguni	Bapedi Damara Nguni	Bezuidenhout Dohne Merino Dormer Dorper S A Merino S A Mutton Meatmaster Van Rooy			Venda
Developed	Imported		Developed	Developed	Developed
Bonsmara	Persian		Kalahari	Kolbroek	Koekoek
Drakensberger	Karakul		Boer Goat	Robuster	SA Ross
Huguenot				Windsnyer	

### Cattle Production

Indigenous cattle breeds, collectively the Sanga with characteristic servico-thoracic humps, were multipurpose, providing sustenance, wealth, or status and an integral part of social and spiritual customs. The Afrikaner cattle (Sanga) impressed the settlers with their size, thriving on harsh, sparse vegetation of the semidesert, their fertility, calving with ease, and raising healthy calves. They were milked for household use, were excellent draught animals, and were tolerant of ticks, endoparasites, and the endemic diseases. Nguni cattle of the eastern regions were less impressive mainly due to a smaller size, but were equally endowed with the survival traits for the African bush.

Afrikaner cattle were the original backbone of the cattle industry. However, the European settler-farmers were of the opinion that the indigenous cattle, despite their adaptive attributes, did not exhibit beef and dairy production traits as those of European breeds. The importation began of beef, dual-purpose, and dairy breeds. Imported breeds had been selected for specific productivity traits in their respective regions of origin. In Africa, these breeds had to deal with the environment and endemic diseases. This gave rise to important changes in the beef and dairy industries: Genetic selection within breeds for hardiness and productivity; crossbreeding with indigenous cattle, mainly the Afrikaner breed to consolidate hardiness and productivity traits. The process leads to scientific research and the development of veterinary medicines and prophylactic treatments to control parasites and endemic diseases.

The observation of Bosman (1932) that “the increase in Native-owned stock is significant as it is related to a decrease in cattle losses due to disease, drought and exposure, and this one would expect to influence favourably the European-owned stock rather than the less cared-for Native-owned stock. The opposite is, however, the case,” placed the inherent value of the indigenous cattle in perspective. Schoeman (1989) concluded that indigenous cattle may be more production efficient than exotic breeds. The genetic admixture of the Sanga is a basis for their genetic improvement (Makina et al., 2016), while their value in composite breeds has been widely demonstrated as in the Bonsmara breed (Bosman et al., 2017), which has emerged as the most numerous beef breed.

Importing European cattle breeds, developing composite breeds such as the Bonsmara cattle (Webb, 2009), and importing composite breeds from Australia and Brazil continued to expand the cattle population diversity. Breed societies were established to enhance a breed’s image through cattle shows and performance data. State initiated performance-recording schemes, including carcass and meat, and milk quality assessments, and artificial reproduction technologies were launched, with discernible consequences of improved production efficiency in meat and dairy production. Regrettably, too large a number of the beef industry on both freehold farms and communal systems do not benefit from performance recording. Nonparticipation in performance recording due to reluctance, nonservices, education, and socioeconomic factors especially in the deep rural areas could be a major hindrance to developing and maintaining sustainable animal production.

Semi-intensive and intensive systems can drive production efficiencies. Feedlots remove cattle from the extensive rangeland for custom feeding; dairy system combines genetic selection with specified nutrition. Improved general husbandry increases yields per lactation and fewer lactations per cow.

Intensive and semi-intensive systems have an added cost of reliance on off-farm feeds and services. Intensification has raised the stakes on animal welfare. Veterinary services have increased to deal with production-related pathologies. In these systems, environmental stressors can have debilitating, disruptive effects on the livestock’s endocrine system (Bova et al., 2014). In addition, metabolic modifiers such as Zilpaterol hydrochloride approved in South Africa in 1997 for use in beef cattle (Montgomery et al., 2009) and recombinant bovine somatotropin (r-bST) for use in dairy cattle (Erasmus and Webb, 2013) influence the animals’ physiology, and if not managed correctly, may have impacts on the environment.

Expansion of the beef industry is attainable, particularly with extensive production. This would increase the sustainability and the amount of product produced. Economic factors hamper the expansion of dairy production; the total number of dairy farms has decreased with an increase in the size of farms. Efficiency in the beef and dairy industries is improving, which is driven by supplementary and designer nutrition, constant review of genomics, physiological and morphological observations such as claw quality (Van Marle-Köster et al., 2019), and general animal husbandry. The average size of a dairy herd in South Africa is 459 cows in milk, placing South Africa second on an international comparative listing, with Saudi Arabia at the top with 7139 cows and New Zealand third with 416 cows followed by Australia with 274 cows (IFCN, 2019).

### Small Stock

Sheep and goats have similar historical developments to cattle. Sheep and goats are primary pastoral animals in term of numbers and the extent of the range occupied. Indigenous sheep and goats are still farmed with breed societies supporting the efforts. Fat and lean-tailed sheep breeds are small framed with localized fat deposits and had short-haired pelts with minimal wool, excellent morphological and physiological traits for enduring the environmental rigors. The products were mutton, fat rendered for food and industry and skins for various manufactured items. However, South Africa required a sheep breed that has excellent meat production traits of carcass quality, fertility and suitability for arid conditions. The Dorper was developed from exotic British Dorset Horn and the Blackhead Persian breeds (Hugo, 1966). The Dorper is highly successful locally and internationally.

The importation of Escorial Merinos in 1789 changed the sheep industry (Hugo, 1966) that was followed with further importations of Merino sheep from Saxony, Escorial, and Negretti. The environment of the Karoo region produced excellent fine wool. Today, the S. A. Merino and derivatives such as the S. A. Mutton Merino constitute the largest proportion of sheep followed by the Dorper.

Unimproved indigenous or Savannah goats of varying sizes, coats, and patterns abound in the rural African areas. The Boer goat, a descendant by selection of its carcass size and prolificacy with a distinct red head and white body (black heads also occur), is an international standard for a meat goat (Casey and van Niekerk, 1988; Van Niekerk and Casey, 1988), with desirable female reproduction traits (Greyling, 2000). The milk goat industry is relatively small producing milk for a select market and goat cheese for export.

The Angora goat, renowned for its high-quality mohair, is the jewel in the crown of the goat industry. Mohair production is solely on extensive dry, shrub-regions. In some instances, farmers keep their kidding does on pastures irrigated from groundwater. The industry is internationally well-established producing 60% of the global clip. The first genetic material by way of a pregnant doe arrived in SA in 1838 from Turkey. Importations were sporadic to 1880 (Hugo, 1966). Production, genetics, and product quality traits are well researched in South Africa, indicating the emphasis on developing the industry further (Visser and Van Marle-Köster, 2014; Snyman, 2020).

As with cattle, the state, breed societies, and structured industries sponsor breeding and selection, research into optimum nutrition, and marketing of primary and secondary products. Expansion of small stock in numbers is limited due to the carrying capacity of the natural rangeland, which varies by climatic conditions.

### Nonruminant-Intensive Systems

Optimum animal husbandry practices for the species determine the approach to developing intensive sustainable animal production. Determining factors are keeping large numbers of animals on small areas, either on open pastures or in corals as with ostriches, sheltered housing that could be totally or partially enclosed as with swine and chickens, or a combination of open areas and housing. Crocodile farming requires an open pool and lounging area.

Highly mechanized intensive animal enterprises encompass challenges of 1) providing scientifically determined feed for all stages of development, 2) high-density populations that can result in induced social stress and the risk of pathologies, and 3) concentrated, mechanized, waste management. Feed, from production to feeding, is an off-farm enterprise as a feature of the Holocene Era where humans increasingly develop additional practices to support what would have been natural processes. Even free-range commercial systems are not entirely free-range. Free-ranging poultry and swine are present in villages and rural areas, where swine present a threat of zoonotic pathologies, in particular *Taenia solium* and *Cysticercosis*.

The swine industry does not rely on indigenous swine that are characteristically early-maturing, fat animals. These animals have the indigenous adaptive and survival traits, but growth rates and feed conversion do not match those of developed genotypes, which prevail. The industry is highly organized with ethical standards and class standards for all carcasses that pass through registered abattoirs. Visser (2014) presented a comprehensive overview of South Africa’s swine industry.

Poultry egg and broiler industries are intensive in-door systems with the egg industry accommodating a few semi-intensive systems that are considered free-range production. The industry is based on imported, highly productive genotypes. Housing is either semi-open-sided or entirely enclosed. These systems, as noted with swine, have a high reliance on off-farm feed supplies, and the inevitable waste. Indigenous chickens are kept as scavenging or true free-range survivors. Indigenous chickens have high heat and humidity tolerances (Garces et al., 2001), are fertile, and are protective mothers. Since they are free-ranging, these birds do not suffer the pathologies associated with caged birds such as lameness and cannibalism, though they are pretty competitive. The indigenous poultry are prone to parasites, but weather the inconveniences well.

Egg and broiler production are the fastest expanding sustainable animal production systems, facilitated by the opportunity of many farmers including those in communal farming regions to obtain chickens from centralized breeding and incubation localities. Scientific development is driven by nutrition (macro and micronutrients) and the control of pathologies. Commercial genetic material for high producing genotypes is produced through international breeding companies. Genetic material for locally developed breeds that are mainly dual-purpose types is of local origin.

Ostrich and crocodile farming are classed in the exotic leather industry. Ostriches are true indigenous game-poultry and remain wild continually expressing their temperaments, though they may become habituated. South Africa is renowned for its ostrich industry. However, competitive production is increasing in many countries. The industry is semi-intensive. Breeding clusters, usually a male to two females, are kept in open corals, the eggs are hatched in incubators, and the chicks are kept in houses or on pastures. Growing birds are kept in corals. Feed is mostly off-farm with the exception of young birds on pastures. Waste management is not considered problematic since the typical production regions are dry as semidesert or are located in subtropical seasonal rainfall regions. Ostriches produce feathers for the fashion market and industrial use, highly valuable hides, meat, and byproducts such as shell-based items.

Ostriches are prone to a number of diseases, especially in the high-density populations and chicks (Verwoerd, 2000). The industry fluctuates: it relies on exports and diseases often cause embargoes. Recent research in the crocodile industry has focused on nutrition (Bland et al., 2021, personal communication), incubation, quality of the hides, and pathologies (Dzoma et al., 2008, Hoffman et al., 2000, Huchzermeyer, 2002).

Expansion of intensive sustainable animal production appears abundant for the poultry industry. The relative low per capita consumption of pork due to cultural preferences limits expansion of the swine industry. The ostrich industry has shrunk severely over the past decades due mainly to disease related prohibitions on imports. The crocodile industry is relatively small, but is in a growth phase.

## Managing the Production Environment

The extensive production environment ranges from high rainfall, high carrying capacity to the opposite with scrub forage. Managing this vast range requires expertise in rangeland management, soil conservation, agricultural meteorology, and animal husbandry. Managing water similarly requires water conservation, supply, and quality management applications (Casey et al., 1996, 2016; Scholtz et al., 2013).

## Managing Health and Welfare

Managing health and welfare in all sectors of the formal livestock industry is done by adherence to the prescribed veterinary regulations, and with the aid of a veterinary inspector in the communal and small-scale farming regions. Each of the industry sectors such as the red meat, wool, mohair, dairy, swine, and poultry producers has codes of conduct for animal health and welfare, and product quality. Various legislations regulate the traceability of livestock and livestock products. Legislation enforces the control of infectious, vector-borne parasitic and zoonotic diseases.

## Greenhouse Gas Emissions

South Africa is the 14th largest contributor to greenhouse gas emissions (GHGe) mainly due to its reliance on coal (www.carbonbrief.org). The country is committed to monitoring and reducing GHGe, having endorsed the United Nations Framework Convention on Climate Change (UNFCCC) in 1997 and the Kyoto Protocol in 2002, and ratified the Paris Agreement on Climate Change in 2016 (www.environment.gov.za).

Monitoring and quantifying GHGe are intended to identify sources with the further intention to reduce the emissions, thereby contributing to reducing the effects they have on the global climate. Quantifying GHGe is beset however by uncertainties (Jonas et al., 2019). These include as adapted from Jonas et al. (2019), accurately and precisely accounting for emissions in space and time, complying with emission reduction commitments, considering risks of temperature targets, evaluating mitigation and adaptation strategies, and potentially trading emission permits. It is important to deal with the quantifications according to a structured framework to alleviate the uncertainties, as these authors propose (Jonas et al., 2019).

The GHG CO_2e_ in South Africa reported in the Third Assessment Report, 2000–2015 (GHG NIR SA, 2015) excluding agriculture, forestry, and other land-use data (AFOLU) increased from 2001 (442,247 Gg) to 2015 (544,746 Gg) by 23.18%. Including the AFOLU sector, the net increase was 20.28%. The differences, which could be ascribed to mitigation by the AFOLU as a carbon sink, were 2.94% in 2001 and 5.13% in 2015. Managed pastures, rangelands, forest, and crops are well-recognized carbon sinks that could mitigate a proportion of national GHGe, and more specifically the AFOLU emissions. The issue remains that AFOLU emissions are a combination of emissions from animal and tillage plus that of services. The carbon emissions from animals and tillage are cyclical and largely in balance. Services emit fossil carbon that in essence is not cyclical.

### Enteric Fermentation

The enteric fermentation digestive processes by which plant material is broken down in herbivores, and by the cecum digesters, equines and swine, produce methane as a byproduct. Estimates of enteric fermentation CO_2e_ for the animal sectors listed in Table 3, were derived from applying the IPCC 2006 equation 10.20 and the values derived by Du Toit (2013a–d) and Moeletsi and Tongwane (2015). It is important to note that the report presents the comment that the differences between data can be ascribed to nonstandard methodologies to calculate CO_2e_. South Africa has not developed their own estimate equations of enteric fermentation emission for these animals and relied on Australian equations, since the countries have similar animal production environments (GHG NIR SA, 2015).

**Table 3.** Trend and relative contribution of the various livestock categories to the enteric fermentation emissions between 2000 and 2015

	Emissions (Gg CO_2e_)		Change 2000 to 2015		Share of enteric fermentation (%)	
	2000	2015	Values	%	2000	2015
Cattle—dairy	2,470	2,272	−198	−8.0	9.26	8.78
Cattle—nondairy	18,348	18,233	−115	−0.6	68.80	70.45
Sheep	3,801	3,391	−410	−10.8	14.25	13.10
Goats	907	755	−152	−16.8	3.40	2.92
Horses	102	119	17	16.7	0.38	0.46
Mules and asses	34	36	2	5.9	0.13	0.14
Swine	44	40	−4	−9.1	0.16	0.15
Other (game)	961	1,036	75	7.8	3.60	4.00
	26,667	25,882	−785	−14.93	100.00	100.00

Source: GHG NIR SA (2015).

The GHG NIR SA (2015) report notes that “In 2015 the enteric fermentation category contributed 25 881Gg CO_2_e (Table 3). Non-dairy and dairy cattle contributed 18 233 881Gg CO_2_e (70.45%) and 2 727 Gg CO_2_e (8.8 %) respectively to the enteric fermentation category. Emissions from horses, mules and asses, and other (game) increased between 2000 and 2015, while emissions from all other livestock declined. The largest decline was seen in the enteric fermentation from sheep, which declined by 10.8 % over the 15-year period. These emission trends declined by 2.9% since 2000 to 2015.”

The GHG NIR SA (2015) report did not include poultry, which left out a livestock sector that constitutes 39 % of the gross value of animal products. Poultry however have comparatively low enteric emissions, which were placed in perspective in the data of Monteney et al. (2001) cited by Dunkley and Dunkley (2013) in which the percentages of the combined emissions were respectively dairy cattle 56.5%, beef cattle 29.6%, swine 11,7%, and poultry 4.4%. The fraction of poultry enteric fermentation emissions becomes a significant contribution to national enteric fermentation emissions from livestock considering the numbers of birds that constitute 39% of the gross value of animal products.

### Comparative Data

Comparative data suggest that South Africa’s animal GHGe (GHG NIR SA, 2015) are within the reported ranges. If the CO_2e_ for cattle is taken as the average emission kg CO_2e_/kg, the value for South Africa is 16.26 kg CO_2e_/kg, which compares well with the 16.25 kg CO_2e_/kg given by Dunkley and Dunkley (2013), and for swine, 3.14 kg CO_2e_/kg compares well with the cited values. The similarities in estimated kg CO_2e_/kg on national or regional scales stand to reason since the equations are the IPCC (2006) versions. A review of the equations for the three Tiers could result in different outcomes. However, the same dilemma of the equations being the common denominator would occur. Thomas et al. (2011) noted the involvement by the animal production industry is important in developing new technologies for quantifying GHGe.

Differences could result when the emissions are related to different animal farming systems within a sector. Hagemann et al. (2012) as a sequence to Hagemann et al. (2011) noted that estimates of GHGe as a consequence of milk production, do not account for the diversity of systems. Numerous authors have raised the point of accounting for diversity.

A series of articles that presented the estimates of direct GHGe of the four animal sectors of the beef cattle, small stock, game and monogastric industries (Van Niekerk and Hassen, 2009; Du Toit et al., 2013a–d; Hassen et al., 2015), laid a foundation for subsequent analyses of animal GHGe. The authors had selected Tier 2 methodologies quote IPCC (2006) “that requires detailed country-specific data on gross energy intake and methane conversion factors for specific animal categories. The Tier 2 method should be used if enteric fermentation is a key source category for the animal category that represents a large portion of the country’s total emissions.” Tier 2 offers countries the opportunity to develop methodology specifically for national, regional and sectoral circumstances in order to minimize uncertainties of GHGe.

Two South African studies on GHGe on production practices on dairy farms (Reinecke and Casey, 2017) and a comparison of beef and dairy practices (Tongwane and Moeletsi, 2020) emphasized the need to scrutinize production practices. The former authors reported a range of GHGe in CO_2-eq_ among dairy farm management systems with various methodological approaches. They recommended more detailed equations to estimate emissions as a tool to improving the associated environmental impacts. In terms of format, detailed non-linear equations delivered apparent biologically realistic emission values. Linear equations showed larger prediction variation of GHGe. They concluded that the accounting methodologies applied to predict GHGe could be applied across dairy farm management systems to quantify the carbon footprint of dairy production in South Africa.

Tongwane and Moeletsi (2020) reported dairy cattle emitted more enteric CH_4_ than communal subsistence and commercial beef cattle. In relation to the respective populations, the commercial beef sector accounted for 48 % of enteric CH_4_ emission, subsistence cattle 36 % and dairy cattle 17 %. Further, relating to sustainable animal production, they noted apparent improving cattle production efficiencies indicated by emission factors and emissions per energy-corrected milk and animal carcass weight. Both sets of authors recommended that monitoring GHGe could be useful to monitor production efficiencies. Improving production efficiency would reduce GHGe (Scholtz et al., 2013). However, due to limited resources, socioeconomic and political circumstances, a large part of the livestock industry is faced with overriding issues of survival, which would require concerted efforts to transform production practices.

## Policy Framework

South Africa’s governance framework is based on a Constitution that prescribes three levels of executive authority: national, provincial and metropolitan. National authority is in many cases devolved to the provinces, for example, veterinary services nationally resides under the national department, but devolved authority permits provincial departments to deal with provincial affairs.

The following selection of legislation applies to the topic at hand. Since the legislations are amended periodically, the basic reference document is cited. Amendments may be sourced at www.gov.za:

Agricultural Product Standards, Act 119, 1990

Agricultural Research, Act 86, 1990

Animal Diseases, Act 35, 1984

Animal Health, Act, 7, 2002

Animal Identification, Act 6, 2002

Animal Improvement, Act, 62, 1998

Climate Change Bill, 2018

Conservation of Agricultural Resources, Act 43, 1983

Fertilizers, Farm Feeds, Agricultural Remedies and Stock Remedies, Act 36, 1947

Genetically Modified Organisms, Act 15, 1997

Meat Safety, Act 40, 2000

Medicines and Related Substances Control, Act 101, 1965

National Climate Change Adaptation Strategy (Draft), 2019

National Environmental Management: Air Quality Act 39, 2004 (GHGe)

National Environmental Management: Biodiversity Act 10, 2004

National Water Act, 36, 1998

All natural scientists in practice are required to register with the South African Council for Natural Scientific Professions (Act 27, 2003), and all persons practicing in the veterinary field register with the Veterinary and Para-Veterinary Professions (Act 19, 1982).

## Conclusion

South Africa maintains a healthy attitude toward sustainable animal production systems that is supported through legislation, commitments of industry sectors, and private- and government-sponsored research. Against the background of South Africa’s colonial development, the animal production industry supports socio-economic and political adaptations to changing circumstances. Sustainable animal production in all aspects will only result if all participants in animal production are supported and empowered.

## About the Author

**Norman Casey**, emeritus professor in Animal Science at the University of Pretoria, graduated BSc (Agric) and MSc (Agric) at the University of Natal and attained a DSc (Agric) at the University of Pretoria. He is a registered professional animal scientist with the South African Council for Natural Scientific Professions, Pr. Sci. Nat. (Anim. Sci.). He practices in livestock production physiology, although his academic work extends into associated fields, including tertiary education in the broader field of livestock production. He has held numerous offices in academia and in the scientific community including president of the statutory South African Council for Natural Scientific Professions and of the World Association for Animal Production.
